# American Medical Association

**Published:** 1891-10

**Authors:** 

**Affiliations:** Chicago, Ill.


### AMERICAN MEDICAL ASSOCIATION.

*Wednesday, May 6, 1891.—Morning Session.*

### SECTION OF ORAL AND DENTAL SURGERY.

(Continued from page 631.)

Dr. George W. Whitefield read a paper on "Pathological Conditions produced by Galvanic Action between Dissimilar Metals in the Treatment of Caries of the Teeth," and then illustrated some of the points in his paper by an exhibition of electrical apparatus. (For Dr. Whitefield's paper, see page 671.)

*Dr. Williams.*—In this experiment one element is amalgam. What is the composition of that amalgam?

*Dr. Whitefield.*—Ordinary commercial amalgam. I don't remember its composition.

*Dr. Williams.*—One point that I can see in this paper is that what we have practised can be verified by strict scientific experiments. I will simply say one or two words. I have noticed from general observation, and am convinced, that amalgam with zinc is more liable to produce galvanic action than without zinc.

Another point is that in several cases where there has been decided galvanic action, I have found that the galvanic action was less, or not existing at all, by combining as large a proportion of gold-foil with the amalgam, as possible. It is on the theory in a general way that by mixing the gold with the amalgam, if there is gold filling near, it lessens the difference in conductivity between the metals and so lessens the galvanic action; that is my theory, practised for years.

*Dr. Marshall.*—I am interested in this paper and also in the experiments, and have this to say of some experiments I have seen. Dr. S. P. Palmer, of Syracuse, New York, demonstrated fifteen years ago, or rather, I should say, proved very conclusively, that there was not only action between tooth-structure and metal fillings, gold and amalgam plugs, but also between gold and tin and between tin and amalgam, but of course the action was not so great between the latter as between gold and amalgam. He also went considerably further than this in his experiments. He proved conclusively that a galvanic action was established between any two dissimilar materials when moistened with a solution of chloride of sodium, or saliva, producing a distinct deflection of the needle of the galvanometer. Another experiment seemed very strange at the time; for instance, he was in the habit of taking two foods which we eat in pairs, as the most palatable, such as ham and fried eggs, a piece of mutton and tomato sauce, a piece of turkey and cranberry sauce; in fact, he followed it all through the foods which we naturally eat in pairs, and found that by moistening these with saliva that there was a deflection of the needle as a result. He then made this suggestion, that on account of this peculiar galvanic or electrical action present between certain dissimilar bodies or kinds of food, zest of taste or relish is given by the decomposition of the saliva, produced by the electrical action between the foods.

"How many of us ever tasted water as good as that of the well on the old farm?" Dr. Palmer would ask. "What did you drink out of, the tin dipper? The contact of the metal dipper with the moist lip produces a galvanic current. That is the solution of it." I am inclined to think that there is a good deal in this. I just mentioned this to show what has been done experimentally in this very direction by Dr. Palmer. Most of you know that Drs. Palmer, Flagg, and Chase were the three men who made up the new-departure trio. Their idea was that gold was not always the best material to use in the filling of teeth; that sometimes amalgam was better than gold itself, on account of the low galvanic condition which existed between amalgam and some of the baser metals and tooth-structure.

*Dr. Williams.*—There is one point in regard to Dr. Palmer's idea that I would mention which I think is a little wanting, because I consider as a general thing, and certainly the poet so mentions it, that the most delicious flavor of water is from an old oaken bucket.

*Dr. Marshall.*—Dr. Palmer in his experiments also proved that just as soon as the surface of the amalgam plug became thoroughly oxidized, electrical action ceased. It was only in such cases in which the plug remained bright that this action continued. You all have no doubt noticed that when an amalgam plug has been placed in the tooth in contact with a gold plug, in a few days oxidation commences; and it used to be thought that we must leave a bridge or partition of dentine or tooth-structure between the two plugs, but we found after a time that the tooth at the cervical margins of the plugs was very soon dissolved away, and that when you allow them to come in contact this condition did not occur.

*Dr. Custer.*—I think you might gather from this that the amalgam which oxidizes most easily will be the best.

*Dr. Whitefield.*—I made a statement that where amalgams oxidize rapidly they are best; zinc will never oxidize thoroughly. I will state the following case: A patient came to me for treatment, and while under my care she complained that the fillings that had just been inserted were giving trouble. It immediately called my attention to the electrical action. I removed the amalgam filling and in a short time all the trouble stopped. Using amalgam in preference to gold in some cases has been suggested. In some cases they need a kind of whip. There needs to be some kind of a stimulant.

*Dr. Williams.*—I would like to say that I hope that Dr. Whitefield will try a suggestion of mine, which is to mix as much gold as possible and then put it under test. I hope he will try it practically, scientifically, and mathematically; not try to find a certain thing, but try to see what he can find.

Dr. Marshall's paper was next read. (See page 690.)

*Dr. Williams.*—Mr. Chairman, I have a few words to say, and that is that this paper seems to me to be a very sound and rational paper in every respect. There is a point of originality in the cause of the accretions which it strikes me is quite new. Another point that I fully concur in is that the local treatment, whether by ploughing with instruments or by application of various medicaments, will not produce the preventive action we want.

*Dr. Noble.*—I was very much interested in one or two points in the paper, and one was the source of this deposit that we find frequently in these deep-seated troubles. I know that we want to seek for something besides a local cause. I was very glad to see a proof of Dr. Marshall's ability in that direction. I have not much to say, because this is rather a new thought to me in looking for these troubles; but I shall in the future try to observe some facts suggested by Dr. Marshall's paper. I have not the slightest doubt but they are derived from correct observation. I think we must look to the system far more than we have in general treatment. It is rather in the general system than in local causes that we should expect to find many of the most marked and deep-seated troubles. I rejoice to see a paper with the perfection with which this was placed before us, and shall read its publication with a great deal of interest, and try to see if the statements made by Dr. Marshall cannot be verified by observation in my own practice.

*Dr. Whitefield.*—I would like to say a few words about a case that bears on this subject. I have in mind a patient who came to me seven years ago with almost perfect teeth, but her gums were highly lacerated. The explanation she gave was that she used to have a desire to probe her teeth continually, and thought that caused the trouble. At the time I did not understand how to treat her; but later on I found out the lady was suffering from a rheumatic diathesis, and every once in a while this would occur. As I knew her better, I now can recall that she would have this trouble about the time she would have the rheumatic trouble. She was past the middle period of life, and had almost perfectly sound teeth. She was very cleanly, and I never could find a particle of tartar about her teeth. I am therefore confident that Dr. Marshall's deductions are correct.

*Dr. Daniels.*—I would not attempt to discuss a paper like this without some preparation. I certainly think that in all of these papers there ought to be some time allowed for criticism. I have no doubt that Dr. Marshall has given a great deal of thought to it that will lead in the right direction, and we will learn more about the manner of investigating this very serious trouble. I would like to hear others on the same subject.

*Dr. Taft.*—Gentlemen, I have had a great many ideas on this subject in my time, some of which proved afterwards to be not very good. In reference to these particular deposits upon the root of the tooth, in connection with the suppuration that takes place between the root of the tooth and its surrounding tissue in the disease of the periosteum, or tissue surrounding the cementum, it is found in various forms, as you all know. Sometimes it is more dense than ordinarily. Sometimes there is a considerable amount, in thick clumps, and in some cases largely distributed over the roots of the teeth. I think it is always of a darker color than ordinary salivary calculus. There are a great many varieties, and it will be interesting to follow them out and the paper leading in that direction. It would be an interesting question to decide fully by induction the action of these various presentations of this trouble. The deposit is sometimes very rough on its surface, in other instances quite smooth. However it may be in these respects, it presents a surface to which the tissue can never unite, and while it remains there will necessarily be a diseased condition. That it comes from the saliva there is no evidence whatever, as indicated in the paper. It was formerly assumed by many that it occurs in the same way that ordinary salivary calculus does; but there are so many counter-indications that this theory cannot be considered as correct, and we must look for the trouble elsewhere. I have no doubt this material is a deposit from the blood. Where this disease takes place, how this disease begins, perhaps, is not so easily explained; that is, how the disease occurs in connection with this condition of affairs. As a matter of course, there must be an irritant in the part before any deposit takes place; that this deposit is a primary thing in this respect I do not believe. I think it is a consequence of the diseased condition that existed before any deposit at all was formed. What is the irritant or cause of this disturbance in the first place I will not now pretend to say,—only that it is at first a point of deposit, a point upon which vicious matter is retained, that becomes more and more vicious as it remains. Dead material that comes into such a pocket or space will be more likely to be retained on this roughened surface. It occupies the place that living tissue ought to occupy, and then again where it is rough, and sometimes it is found very rough, it consists of little nodules all over the surface. Allowing the tissue to come in contact with it will create irritation. There will be irritation induced by pressure against it, and when there is a rush of blood to the part an enlargement of the vessels in the immediate vicinity thus occurs. This sensation has been referred to by Dr. Marshall that persons desirous of assuaging pain will do so by pressure upon the teeth and that will give relief. By pressure it relieves the distended vessels, and then by letting up again there is a sense of relief.

Well, then, the question occurs, what shall be done? Mere systemic treatment will not do. I do not believe that after this deposit has been made upon the roots of the teeth that it can be removed by systemic treatment. It is true that the system is in a bad condition and may be improved, and whatever may be done in that respect increases the tone and strength, and often relieves the system of embarrassment of this kind, although it will not remove this particular deposit. It is sometimes confined to the end of the root, and there may be very little of a pocket. Sometimes it may be found on teeth on one side, sometimes at the end; in molar teeth it is often in the bifurcation of the roots. Its frequent point of deposit in such locations render it exceedingly difficult to remove, as this material does not readily dissolve by solvents; that is, where there is a large mass of it, it is not easily dissolved. In testing the solubility of this material in sulphuric acid it does not quickly dissolve; so that what should be done with it is a question that is difficult of solution. If there is a pocket running down, it may be removed with a fine chisel-shaped scaler; but what shall be done when there is no pocket, when there is not a large space around the edge of the root? I have, not long since, seen superior molars removed with three well-defined roots, in which at the bifurcation there were large masses of this material. In a case of mine, where the patient was exceedingly anxious to save the tooth, which we always try to do when we can, he had given it treatment both surgically and with applications, but the tooth had to be taken out. Upon the outside of the roots, where they could be reached with a scaling instrument, they were in good condition, but where the surfaces were inaccessible by the instrument, sulphuric acid had been used, but with very little effect. Now, a case of this kind is very difficult to manage.

Another point is, What shall be done in the way of prophylaxis, before any disease has taken place? Can any treatment be employed before other disease commences? Are there any indications, are there any symptoms in the beginning that will aid you, and for which remedial or preventive treatment may be employed? These are points to which attention ought to be given and which we ought to study as thoroughly as possible in order to attain to an effective treatment or management of such cases. When we speak of prophylaxis, I suppose we speak of management of the body in such a way as to divert or prevent disease. What can be done to prevent this, and when that is ascertained what treatment may be employed to prevent or cure this condition? I fancy that we do not sufficiently understand the beginning of diseases of the teeth, and that therefore our prophylactic treatment and suggestions with reference to prophylaxis is quite below what it ought to be, and what it ought to be for the best interest of patients. What light ought we to have on this subject?

*Dr. Whitefield.*—It seems to me that the value of Dr. Marshall's paper is that it does not point out treatment except in a general way. The question we ought to know is whether persons with rheumatic diathesis are more liable to have trouble.

*Dr. Marshall.*—Mr. President, I purposely avoided in my paper any reference to a special method of treatment. My idea was to present a paper on the pathology of the condition; and of course, as the title of my paper indicates, I confined it to the rheumatic and gouty diathesis as manifested in diseases of the peridental membrane. In reply to Dr. Whitefield's question, I may say that I have never noticed any difference between living and dead teeth so far as these particular manifestations in the peridental membrane are concerned. The remarks of Dr. Taft deal very largely with the point brought out in the paper with regard to location of concretions on the teeth and their character. Dr. Noble asked a question which I do not think was fully understood: whether these concretions were found at the apex, or upon the root of the tooth where there was no external opening at the margin of the gum, which proves, I think, my position. I claim such conditions exist as a result of rheumatism and gout,—viz., that the concretions are not of salivary origin. The issue I take with Dr. Ingersoll is that these deposits are not necessarily the result of suppuration, or that lime-salts are deposited as the result of inflammatory conditions. I claim, on the other hand, that the concretions just referred to are the results of a rheumatic or gouty diathesis, and are deposited upon the cementum through the agency of the pericementum, just as concretions are deposited in the synovial membrane in rheumatic arthritis. I have never seen a case which I classify as rheumatic without finding concretions present; but phagedenic pericementitis is not always the result of a rheumatic or gouty diathesis. You will find a great many cases of phagedenic pericementitis in which the roots of the teeth are perfectly clean. To illustrate, and I mentioned this in a discussion some years ago before the American Dental Association at Minneapolis, phagedenic pericementitis is often the direct result of peculiar neurotic conditions of the system. I recall the first case of this character that came under my notice. When the patient came into my hands I tried to find concretions upon the roots of the teeth, but could find none, and I was at a loss to explain the difficulty. I made up my mind, after careful study, that there was a neurotic condition, or at any rate a reflex nervous condition, that affected the general health, and suspected from certain symptoms that she was suffering from some uterine displacement, so sent her to a specialist with the request that he would thoroughly examine the case. He reported that she had an antiversion of the uterus, and replaced it; and in less than three months her mouth was well. All the treatment she had was an antiseptic mouth-wash and the replacement of the uterus by the specialist. I have noticed also other cases in which individuals suffering from diseased conditions of remote organs had this same affection of the teeth. Phagedenic pericementitis is often associated with Bright's disease and diabetes mellitus. Concretions upon the roots are, however, the most common cause that produces this condition of the pericementum.

*Dr. Taft.*—What is your opinion in regard to the presence of these secretions in inflammatory cases?

*Dr. Marshall.*—I claim the concretions are the cause of the inflammation; they are the result of the peculiar diathesis of the system, and not the result of the inflammatory action. We find in the synovial membranes that acid crystals are deposited there, and by their presence cause irritation. We sometimes find associated with these crystals the calcium salts; and the longer the disease has been running the more likely is it that we find this peculiar condition of the concretion; that is, the presence of lime-salts. I have not been able yet to demonstrate by chemical analysis whether or not the concretions upon the roots of the teeth are really made up of urate of soda and lime-salts. I hope some time to be able to make the analysis; and if I get hold of a case in which the teeth must be extracted, and am sure the disease is caused by this rheumatic diathesis, I shall submit it to the best chemist we have in Chicago with instructions to examine it thoroughly and report as to its composition. That is the only way to prove the theory that I have advanced.

*Dr. Whitefield.*—Do you not consider that there may be soreness long before actual deposition?

*Dr. Marshall.*—That is one of the general symptoms of this rheumatic or gouty condition of the system. You will find in scores of cases that the very first symptom of rheumatic trouble presented will be soreness of the teeth. In a great many cases they do not progress beyond the stage of congestion, which simply results in soreness and thickening of the membrane; a great majority, however, of the chronic cases result in exostosis of the roots, and in enlargement of the epiphyseal ends of the long bones. The cases in which there are pus-pockets formed are comparatively small in comparison to those that result in periodical soreness of the teeth with thickening of the peridental membrane.

*Dr. Williams.*—I hope Dr. Marshall will report after he has made the experiments.

*Dr. Marshall.*—Another point I would like to mention is that in regard to the systemic treatment. I indicated in my paper that in a great number of these cases I analyzed the urine, and found that the uric acid was largely in excess. In a case of this kind I would say to my patient, you are living too high, my friend; you will have to cut off your wine and eat no more meat, but be a vegetarian; and just as I restricted him in these things his symptoms would improve. This class of cases are just as amenable to systemic treatment as the ordinary cases of inflammation of the joints. If you take it early enough you can control the symptoms, and it was this fact, as I mentioned in my paper, which led me up to the thought which I have presented with regard to the origin of many of these affections.

*Dr. Whitefield.*—The value of your paper lies in this last point.

Here Dr. Taft read his paper on "Care of the Teeth."

*Dr. Whitefield.*—The subject treated of in Dr. Taft's paper is one that interests me very much. I have always thought that a man's position depends very much on the way he regards himself. A dentist may be what I may call a scavenger, or he may not, just as he pleases. I certainly would never do any of this kind of work. If a man comes to me with his mouth filthy, I tell him, in as delicate a manner as possible, to go home and cleanse his mouth; and if this is done rightly he will not take offence. I have seen cases where the mouth was so filthy that I would not handle it under any circumstances. I point out to them the harm they are doing their teeth by letting them get in that condition. I find in most cases that when such people come back they have their mouths in good condition; they have thought about what I said. I impress upon them this point, that if they will do their part I will do mine; otherwise I will not do their work. I charge good fees, and my patients expect to have good work done. If they follow my instructions my work will stand and will be a credit to me. I think this matter should be emphasized more than it is by every dentist.

*Dr. Williams.*—I am very glad that Dr. Taft read that paper, because he refers so strongly to the necessity of dental hygiene and about the frequent neglect of dental hygiene. How often do we find teeth fail, even when well filled, because the general health of the mouth is neglected. Why is it? I think I can point out part of the reason why it is. It is because a man calls himself simply a dentist, and thinks that all he has to do is to attend to a particular tooth. The fact is that he ought to be qualified so as to take just as good care of the health of the mouth. We should be orists. That is the proper name for an educated practitioner. As Sir Morrell Mackenzie says, the mouth is the gate of life; we stand guard over that mouth, and not simply over the posts, or whatever you call them. We take charge of the whole mouth. If we have a pride in considering ourselves orists, I think we will naturally take a higher stand-point of vigilance than is generally adopted.

*Dr. MacNaughton.*—The university from which I have the honor of being graduated impressed on the students that we have the right and privilege to charge whatever we think our services are worth; one of these services is to cleanse the teeth perfectly. The perfect cleansing of the mouth of a patient often does them more good than anything we can do for them.

*Dr. Whitefield.*—I thoroughly agree with the lady. What I meant to say was, that where I could induce my patients to cleanse their mouths themselves I would do so.

*Dr. Taft.*—The conservation of the health of the mouth is a great matter; the preservation of it in the best possible condition to resist diseases of the tissue. The mouth is the territory of the orist; it is the territory which is committed to his care, and he must do whatever he can to promote its best interests, and he ought to be able to know and understand the conditions and whatever is necessary to improve them. It is vastly better to preserve teeth and the mouth in a state of health, usefulness, and comfort, than it is to restore them after the incursions of diseases; and this is just the point I make, that this matter is overlooked. You will often treat a tooth to secure a good condition for life perhaps, whereas, if neglected, ruin would ensue so far as the tooth is concerned. It is to arouse attention to this subject that I have prepared this paper.

Dr. Williams then gave a brief epitome of his paper, "Remarks on Incipient Necrosis and Caries," there not being time to read it; after which the session was adjourned *sine die*.

John S. Marshall, M.D.,
*Editor.*

Chicago, Ill.

